# Science Diplomacy for Climate Action and Sustainable Development in Latin America and the Caribbean: How Important Is the Early Career Perspective to New Governance?

**DOI:** 10.3389/frma.2021.657771

**Published:** 2021-09-09

**Authors:** Palmira Cuellar-Ramirez

**Affiliations:** ^1^ Division of Health, Biological and Environmental Sciences, Universidad Abierta y a Distancia de México, Mexico, Mexico; ^2^ Latin America Network of Atmospheric Sciences and Meteorology, Mexico, Mexico; ^3^ Faculty of Sciences, National Autonomous University of Mexico, Mexico, Mexico

**Keywords:** science diplomacy, sustainable development, LAC region, science-policy interface, ECRs

## Abstract

Science diplomacy and science–policy interfaces are tools that science has to address the biggest challenges that the world faces today. The scientific community needs to develop the capacity to bring this scientific knowledge to society and decision-makers for the purposes of new governance of the Earth System and thus a more resilient society. Climate change is one of the most challenging issues the world is currently facing, and the Latin America and Caribbean (LAC) region is highly vulnerable to its consequences, as it is expected to exacerbate environmental, social, and economic problems in the LAC region. In this context, and as an emergency call to address the climate crisis with the latest available science in the region, this paper collects a series of examples of the progress, best practices, gaps, challenges, and solutions. We do so from the perspective of Early Careers Researchers (ECRs) and undergraduate and graduate students, highlighting what we are doing to engage scientists in society–policy–science interaction for the sustainable development agenda and climate action in Latin America and the Caribbean.

## Introduction

Science diplomacy is a growing cross-disciplinary field. The United Nations Educational, Scientific, and Cultural Organization (UNESCO) defines science diplomacy as a tool to achieve foreign policy but also to promote peace and sustainable development using science as a process and communication (UNESCO, 2017). The Madrid Declaration on Science Diplomacy defines science diplomacy as a series of practices between science, technology, and foreign policy (S4D4C, 2019). However, the New Frontiers in Science Diplomacy publication defines three important dimensions of it: science in diplomacy, diplomacy for science, and science for diplomacy (The Royal Society- AAAS, 2010).

All of these definitions have in common that science diplomacy focuses on scientific solutions, expertise, resources, and tools towards an international effort to solve international problems, including biodiversity loss, climate change, environmental degradation, and public health issues (Haynes, 2018; Tolentino, 2020).

In the climate arena, international agreements such as The Paris Agreement are the best examples of international collaboration for concrete climate actions. The Intergovernmental Panel on Climate Change (IPCC) is the scientific body of the United Nations and the World Meteorological Organization (WMO) charged with examining and assessing the latest data and state of knowledge to do with climate to aid in making science-based decisions. During climate negotiations, science diplomacy is a push for climate decision-making. (Tolentino, 2020).

For sustainable development, science diplomacy supports countries efforts to achieve the Sustainable Development Goals (SDGs) using science and advanced technology for the common good of humanity to address cross-border cooperation and partnership in sciences and technology (Saner, 2015). The SDGs need multilateral solutions and South–South Cooperation to create knowledge and build a bridge between diplomats and scientists (Echeverria et al., 2020).

But what role do ECRs play in science diplomacy when it comes to addressing global issues? To achieve this issue, we must first define ECRs—Who are We?.

### Early Careers Researchers—Who Are We?

A person who is in the first stages of research can be referred to as an Early Career Researcher (ECR) or Early Stage Researcher (ESR). An ECR in academia is often defined in terms of someone active in research in the first 5 years following Ph.D. completion. (Agnes Bosanquet et al., 2016).

According to the Economic and Social Research Council (ESRC) of the United Kingdom, ECR stages are doctoral, immediate postdoctoral, and transition to an independent researcher (Economic and Social Research Council, ESRC, 2016). Other research agencies suggest that an ECR is someone within 8 years of the Ph.D. degree or a student or scholar who is at the undergraduate, graduate, or postgraduate level (depending on national context) up to 3 years post-Ph.D. (International Standing Conference for the History of Education, ISCHE, 2021).

While there is not a specific or agreed-upon definition, it remains necessary to identify ECRs (Bazeley, 2003). In general, the ECR definition depends on the context, purpose (grants, internships, scholarships, predoctoral or postdoctoral trainees, new faculty, jobs, congress, training, and courses), and other factors (for example age, level of educational training, or work experience). However, “labelling academics who have a promising but as yet unrealised research career as “young” or “new” ignores those who have come to research later in life or later in their academic career” (Bazeley, 2003).

For the purpose of this paper, ECRs will be defined as students and scholars who are at the undergraduate, graduate, or postgraduate level as well as young professionals, practitioners, and stakeholders within 10 years of their latest degree. Age will not be considered, but people younger than 40 and undergraduates will be prioritized.

### Early Career Researchers in Science Diplomacy: Shaping The Future Of Governance

In recent years, science diplomacy has been developed mostly by fellows of different training, course, and workshop projects at leadership institutions and bodies in the Global North. In Latin America and the Caribbean, there are excellent examples of initiatives to develop science diplomacy for ECRs in the Americas, but more are needed (Table 1). There is still a route to consolidate and systematize relevant science diplomacy in the LAC region (Gual-Soler, 2020).

**TABLE 1:** Examples of science diplomacy capacity building institutions, bodies, and training

Global *Global North institutions, bodies, and projects	LAC Region *South–South cooperation and North-South cooperation
● American Association for the Advancement of Science (AAAS)- Washington	● The Science Diplomacy for public managers of Latin America (Bolivia, 2017)
● The World Academy of Sciences (TWAS)	● The South-American Workshop on Government Scientific Advice (Argentina, 2017)
● The International Institute for Applied Systems Analysis (IIASA)	● The Inter-American Institute for Global Change Research projects and initiatives as Science, Technology, Policy (STeP) fellowship program
● The International Network for Government Science Advice (INGSA)	● The Ibero-American Program of Science and Technology for Development (CYTED)
● Earth System Governance Project	● The Latin America and the Caribbean Open Science Forum (CILAC in Spanish)
● Future Earth	● The São Paulo Innovation and Science Diplomacy School (InnSciD SP)
● The European Union Science Diplomacy Alliance by the European Leadership in Cultural, Science and Innovation Diplomacy (EL-CSID),	● Fellowship program of science-policy interface in Mexico City
● Inventing a shared Science Diplomacy for Europe (InSciDE), and ● Using Science for/in diplomacy for addressing global challenges (S4D4C) projects.	
● SciTech DiploHub, the Barcelona Science and Technology Diplomacy Hub	

These programs are mostly focused on ECRs who are in doctoral programs, post-Ph.D., and transition-to-researcher career stages, and in these stages, it would be difficult for many of them to develop soft skills and understand how world governance and politics work. However a lot of them have developed highly specialized research that is predominantly positive since it can be applied to specific problems that require mostly specialized management; nevertheless, by keeping this in mind, science diplomacy opportunities for people in the earliest stages of scientific life is overlooked.

In the undergraduate stage, many students are part of increasingly cross-disciplinary careers and are concerned with the challenges of the next decades, not just from a scientific view but also from that of new worldwide governance. They want to address the most challenging problems, such as climate change, and are looking for inclusive spaces, even if they are not senior researchers yet.

A great example is the 2030 Agenda and the Paris Agreement, which have objectives to mobilize ECRs globally through activism. Nevertheless, it is still difficult for ECRs to have a direct influence on, or be empowered in, the decision-making and diplomacy processes. The roles of ECRs will depend on the mechanisms that exist to foster their participation in each country.

In the case of SDG 13 Climate Action of the 2030 Agenda, one of its specific objectives is to promote mechanisms to increase capacity for climate change in the least developed countries and small island developing States, with a particular focus on women, youth, local and marginalized communities (United Nations, 2015). Then ECRs from the region, mainly from the Caribbean, should have a particular place of climate and sustainable development action in the LAC region.

In 2018, during the second Forum of the Countries of Latin America and the Caribbean on Sustainable Development of ECLAC, the first parallel event promoted and led by youth in science (undergraduate and graduate ECRs), called Interdisciplinary Science for the knowledge Economy and fulfillment of the SDGs, was held to promote the importance of youth.

ECRs from countries such as Mexico, Chile, Colombia, Peru, Venezuela, and Brazil were involved, and representatives of governments, civil society, and other stakeholders were invited. There, a statement was read on how science and cooperation should be in the region for sustainable development. This was accompanied by a previous survey, where 50 young people in science from the region participated.

One of the important findings of the survey was that the young people who participated think that their governments do not invest enough in science. Another important finding was that one of the greatest challenges faced by youth in science is the lack of capacity building. Remarkably, 97% of survey participants agreed that youth are important for sustainable development. They noted that in order to influence decision-makers, they must carry out cross-disciplinary research and get directly involved in projects that have sustainable development as their axis. By doing so, they leave behind the idea of conducting research only in a specific area and instead show a preference for a more holistic vision. However, when they were asked whether they had participated in high-level events for decision-making and diplomacy, the response was 75% negative; for the 25% who answered yes, only 7.1% had participated more than five times.

As an event outcome, then it is imperative that the number of scientists increases in the region. This can occur at scientific research centers, and also via capacity building at the regional level, to increase cooperation between countries and boost those with the greatest deficiencies. Indeed, south-south cooperation is a key to finding regional solutions. “Within the 2030 Agenda framework, it is essential to position natural sciences and social sciences in a joint way to achieve solutions in Latin American countries with a cross-disciplinary vision, where scientific-environmental character and high social purpose are crucial” (ECR Parallel Event LAC RFSD, 2018).

In 2019, some ECRs helped create the first Youth Forum LAC 2030; previous activity of the third Forum of the Countries of Latin America and the Caribbean on Sustainable Development of ECLAC. There, for the first time, an important number of ECRs in Natural sciences at the undergraduate, masters, and recent undergraduate levels throughout Latin America were involved in a process for review of prioritized Sustainable Development Goals (SDGs). They brought a regional and youth perspective, highlighting the SDG 13 Climate Action, making it different from previous events, which featured little ECR participation from Natural sciences in the LAC region.

Because of their participation, some of the ECRs recreated similar processes at their respective national levels, including the Nationally Determined Contributions (NDC) processes in 2020, where ECRs in Natural Sciences, Natural Resources Management, Biology, Earth Sciences, and Environmental Engineering Sciences led the hard negotiation process and the incorporation of the ECR component into NDC updates. Women at the Early Career Stage in Mexico, Costa Rica, and Colombia led important NDC topics such as the ocean, adaptation, youth, and gender on climate action. These processes also were of interest to other regions, UN bodies, governments, and, of course, other ECRs in the Global South and Global North.

Another important success was the ECR participation in the official delegations at the UN Climate Change Conference (COP25), where ECRs supported their delegations in negotiations. The delegations from Mexico, Costa Rica, and Chile featured ECR participation, and they supported complex negotiation on IPCC reports and science highlights as well human rights, capacity building, and article six negotiations. Finally, an important success was the adherence of Mexico and Costa Rica to an Intergovernmental Declaration on Children, Youth, and Climate Action (CERI, 2019). This process of adherence was led by young women at an Early Career Stage, which should mean a great step towards ECRs participation in climate governance and gender equality in science.

These best practices motivated the development of the first program for Latin American early-careers, mid-careers, and late-careers/senior climate scientists interested in climate negotiations. This project is currently being developed by the Latin American Network of Atmospheric Sciences and Meteorology, RedLAtM, which is an independent network led by ECRs. (RedLAtM, 2021). The purpose will be to develop science diplomacy skills, understanding international, regional, and national complexity and how to start a process for evidence-based policy formulation with the most available science in the climate sciences arena.

In March 2021, the second part of Interdisciplinary Science for the Knowledge Economy and fulfillment of the SDGs, the importance of youth in science, was part of the fourth Forum of the Countries of Latin America and the Caribbean on Sustainable Development of ECLAC. The goal was to promote a space for dialogue and brainstorming where ECRs could be in dialogue, express their opinions, and build routes of action for the monitoring of the 2030 Agenda. The primary participation was ECRs from Venezuela, Mexico, Chile, Ecuador, Puerto Rico, and Peru. The three planetary crises, “climate change, biodiversity loss, and ecosystems degradation”, the post-covid green recovery, the importance of vaccine diplomacy, and the role of ECR in the fight against covid-19 in the LAC region were addressed. (ECR Parallel Event LAC RFSD, 2021).

In April 2021, the “Early Career Scientific Youth in the incidence of the update processes on NDCs in Latin America” parallel event was held at the CILAC 2021 forum, with the participation of ECR from Mexico, Costa Rica, Colombia, and part of the government and academic sectors of Peru and Chile. This event was led by ECRs to facilitate peer networking and emphasizing the role of ECRs in updating and implementing the NDCs. Another topic was to discuss whether the NDCs had the best available science to achieve greater climate ambition in the region, highlighting the importance of ECR leadership in the negotiation and governance processes. This space was the first of its kind for the open dialogue of ECRs and NDC processes in the region. (ECR Parallel Event CILAC, 2021).

Finally, it is worth acknowledging the efforts of other youth networks in science, such as the YESS (Young Earth Scientists System) Community, who, with the collaboration of other ECR networks on the global level, brought the opportunity to incorporate ECRs into the IPCC reviews. In that effort, a total of 27 ECR reviewers from Chile, Colombia, Argentina, Brazil, Peru, and Uruguay contributed to the Second Order Draft (SOD) of Working Group I (WGI November 2019-May 2020) and Working Group II (WGII July 2020-January 2021) of the sixth Assessment Report process (AR6). (YESS Community, 2021).

In March–April 2021, *Hub ciencia emprende*, a project led by ECRs, had the first significant online open course for ECRs in LAC Region on science–policy interface,s where ECRs from the region could understand what is science diplomacy and science–policy interfaces. The course was conducted in Spanish. (Hub Ciencia Emprende, 2021).

## Discussion and Conclusion

The ECRs are involved not only in the scientific aspect but also the social justice, the environmental movement, and the new governance breaking structural inequalities. Nevertheless, there are not enough places to participate due to limited spaces in decision making and science diplomacy and a lack of mechanisms to engage them, a lack of sufficient experience, and a lack of knowledge of how policy and diplomacy work. Collaboration with mid-careers scientists, late-careers scientists, and diplomats is needed to address it. “New generations of scientists need to be trained and must be involved in decision-making and policy processes better known as the science-policy interface.” (ECR Parallel Event LAC RFSD, 2018).

It is visible that ECRs are doing science diplomacy work according to their experience, limitations, and knowledge. Although it is desired that people in their mid and late careers who have more scientific skills are those who can influence decision-making based on the best available science; ECRs are reaching towards spaces for participation. We are helping to begin a contrasting dialogue and co-create a science–policy interface as a process that also democratizes science, helps people to create resilience, and builds solid science diplomacy in the LAC region.

ECRs are creating professional support networks and sharing best practices and experiences among peers. The role of these youth networks around the LAC region, as well as the different youth movements, should be incentives for governments to invest in youth as agents of change. (ECR Parallel Event LAC RFSD, 2018).

The gaps identified so far are the lack of support that exists both financially and in terms of capacity building on science diplomacy and science–policy interface for people in the early stages. It is important to mention that all of the efforts previously shared had no specific financial support and have been achieved thanks to the volunteerism and commitment of the ECRs. These situations are a negative indicator, as, without greater support in the future, these spaces could be lost.

Also, some of the challenges that ECRs face, is that part of the scientific community does not accept these practices as science activities or practices that help their professional development, both academic and work; nevertheless, science diplomacy and science-policy interface are components of science to achieve scientific research, scientific cooperation, science policy, and governance for sustainable development.

Incorporating the ECRs in science diplomacy, international negotiations, and science policy is crucial for a new regional integration, consolidating Latin American and Caribbean diplomacy and bridging the scientific gaps between the North and South. Thanks to the fact that the majority of ECRs are pursuing interdisciplinary careers, they can understand and interact with other research areas in subsequent studies applied to global agendas, thus helping their governments and international governance to solve problems of an international nature, such as climate change.

It is important to note that the ECRs, mainly from Earth Sciences and Health Sciences, are in a learning period, studying and investigating the most relevant findings that compromise life on Earth, and they should be the ones leading environmental and social change. Further, researchers must learn to navigate the policy-making system in order to promote the application of their research in policy development (Burton et al., 2019).

Having science diplomacy training at the beginning of a scientific career could help embrace new views and opportunities for people in science, and contribute to new job creations. This would have to be analyzed with the science policy of each country. This helps us build the capacity for science diplomacy—and not only in terms of scientific research. This also includes helping to avoid unemployment rates in the science fields that exist in the LAC region, acquiring tools at the early stages of scientific careers, and finding out how to apply this knowledge for innovation and new governance.

Finally, some of the ECRs come from backgrounds of cultural and gender diversity, which encourages other minorities to be involved in this formulation of public policies based on evidence, such as the indigenous people, women, people with disabilities, afro-descendants, neurodiverse people, and other youth sectors: groups of the population that in Latin America and the Caribbean are highly vulnerable. This important topic will help to address intergenerational, environmental, and social justice in the LAC region.

The ECRs have proven to be aware of the social, environmental, and economic needs of the region, using science as a tool for sustainable peace in our region. Unfortunately, as long as funding is not available, participation cannot be fully formalized, and we could lose the science diplomats of the future in the region.

## Data Availability

The datasets presented in this article are not readily available because it is part of surveys and personal information is protected by law. Requests to access the datasets should be directed to Latin American Network of Atmospheric Sciences and Meteorology, redlatm.contacto@gmail.com, just public results can be shared. Personal information such as name, age, contact information are protected.
